# RNAseq analysis of olfactory neuroepithelium cytological samples in individuals with Down syndrome compared to euploid controls: a pilot study

**DOI:** 10.1007/s10072-022-06500-2

**Published:** 2022-11-17

**Authors:** Lorenzo Brozzetti, Ilaria Scambi, Loris Bertoldi, Alice Zanini, Giorgio Malacrida, Luca Sacchetto, Lucia Baldassa, Giuseppe Benvenuto, Raffaella Mariotti, Gianluigi Zanusso, Maria Paola Cecchini

**Affiliations:** 1 Department of Neurosciences, Biomedicine and Movement Sciences, Neurology Unit, University of Verona, Verona, Italy; 2 Department of Neurosciences, Biomedicine and Movement Sciences, Anatomy and Histology Section, University of Verona, Strada Le Grazie 8, 37134 Verona, Italy; 3 BMR Genomics Srl, Padua, Italy; 4 Department of Surgery, Dentistry, Paediatrics and Gynaecology, Otolaryngology Section, University of Verona, Verona, Italy; 5 AGBD, Associazione Sindrome di Down, Onlus, Verona, Italy

**Keywords:** Olfactory neuroepithelium swabbing, RNAseq analysis, Differential gene expression analysis, Down syndrome, Euploid controls

## Abstract

Down syndrome is a common genetic disorder caused by partial or complete triplication of chromosome 21. This syndrome shows an overall and progressive impairment of olfactory function, detected early in adulthood. The olfactory neuronal cells are located in the nasal olfactory mucosa and represent the first sensory neurons of the olfactory pathway. Herein, we applied the olfactory swabbing procedure to allow a gentle collection of olfactory epithelial cells in seven individuals with Down syndrome and in ten euploid controls. The aim of this research was to investigate the peripheral gene expression pattern in olfactory epithelial cells through RNAseq analysis. Validated tests (Sniffin’ Sticks Extended test) were used to assess olfactory function. Olfactory scores were correlated with RNAseq results and cognitive scores (Vineland II and Leiter scales). All Down syndrome individuals showed both olfactory deficit and intellectual disability. Down syndrome individuals and euploid controls exhibited clear expression differences in genes located in and outside the chromosome 21. In addition, a significant correlation was found between olfactory test scores and gene expression, while a non-significant correlation emerged between olfactory and cognitive scores. This first preliminary step gives new insights into the Down syndrome olfactory system research, starting from the olfactory neuroepithelium, the first cellular step on the olfactory way.

**Graphical Abstract:**

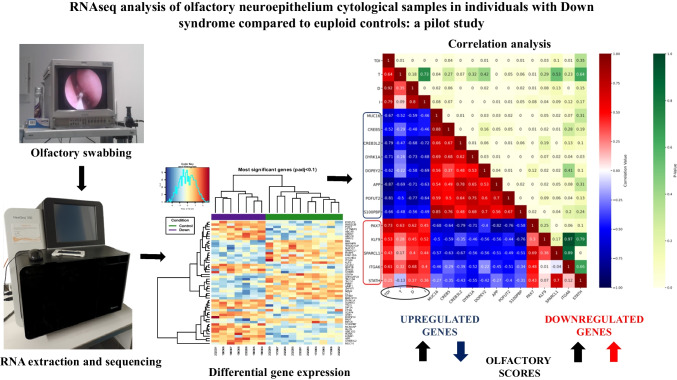

**Supplementary Information:**

The online version contains supplementary material available at 10.1007/s10072-022-06500-2.

## Introduction

Down syndrome (DS) is one of the most common chromosomal abnormalities in live-born children, characterized by well-defined and distinctive phenotypic features. It represents the most frequent form of intellectual disability caused by a microscopically demonstrable chromosomal aberration such as a trisomy of all or a critical portion of chromosome 21 [1–3]. The DS brain is typically reduced in volume since the 13th week of pregnancy and this abnormality contributes to intellectual disability [4]. Neuropathological findings of individuals with DS, such as senile plaques and neurofibrillary tangles, overlap to those of Alzheimer’s disease, and of course occur earlier [5, 6].

These neuropathological hallmarks were also found in cortical brain regions associated with olfactory processing [7–9]. Over the years, research focusing on individual aspects of olfactory function in DS was carried out, describing an olfactory deficit of various degrees [10–19]. The olfactory system can distinguish a very large number of odorant molecules and the nasal olfactory epithelium contains the first neuronal cells, which give rise to the olfactory pathway. Compared to biopsy, olfactory swabbing is a non-invasive and gentle procedure that enables the collection of olfactory epithelial cells in living individuals [20]. In a previous study, we showed that with a single nasal swabbing procedure at the middle turbinate, around 1,000,000 of total epithelial cells are collected. These are composed of olfactory neurons (ONs), which represented the 30% of the total sampled cell population, and by other non-neural cells such as sustentacular and microvillar cells, with supporting/protective function for neurons, not completely unraveled [21, 22].

Gene expression investigations are meaningful to detect expression variations in DS versus euploid tissues in order to understand the molecular effect of genetic overdosage [23]. Previous studies were particularly focused on various human DS tissues or total brains [24, 25], while studies on DS olfactory mucosa are lacking. In this regard, for DS research as well as for other neurological conditions, it could be meaningful to have data from olfactory neuroepithelium samples, other than blood, since this epithelium is composed by neuronal and non-neuronal cells that are easily accessible in living subjects [26, 27]. Therefore, to offer new information on this topic, our aim was to investigate the gene expression pattern of olfactory neuroepithelium samples of DS individuals compared to euploid controls through RNAseq analysis. In view of this, we correlated in both groups the olfactory function with RNAseq results. Moreover, according to our previous work [19], we also made a correlation analysis between olfactory and cognitive scores in DS. Our original approach aimed to provide new insights at DS olfactory neuroepithelium, where the processing of olfactory information starts. This might help to improve the knowledge on the smell impairment in this syndrome.

## Materials and methods

### Subject recruitment

Through the “AGBD Association”' (Associazione Genitori Bambini Down, Marzana, Verona), ten DS individuals were recruited. Exclusion criteria were documented comorbidities able to affect olfactory performance (e.g., recent head trauma, otolaryngology disorders, diabetes, stroke). Three DS individuals withdrew for personal reasons. Finally, a total of seven DS volunteers (*n* = 7; 3 M, 4F, mean age: 23.8 years, age range: 18–33 years) attended the study. Euploid healthy subjects (*n* = 10; 4 M, 6F, mean age: 24.9 years, age range: 22–31 years), matched for age and sex, served as controls. Control group recruitment was done through public announcements at the University of Verona and all subjects recruited were students of the Verona University. All investigations were carried out according to the Helsinki declaration and each subject, or the legal representative, signed informed consent for the olfactory swabbing procedure (Prot.n.28917 June, 15th, 2012).

### Cognitive evaluation

The cognitive datum was an additional information of the DS group, already present at AGBD association, as in our previous work [19]. Cognitive evaluation was performed by an expert psychologist, by means of the Vineland II scale (Vineland Adaptive Behavior Scales-II-second Edition) [28] and the Leiter-R scale (Leiter International Performance Scale-Revised) [29], considering both verbal and non-verbal abilities. Recently, in DS, a high interindividual variability is reported and various cognitive profiles could emerge in the verbal and non-verbal domains [30, 31].

The Vineland-II scale is a valid and reliable method to measure a person’s adaptive level of functioning. It is helpful in diagnosis and in classifying intellectual and developmental disabilities and other disorders, such as developmental delays, and it is organized within a three-domain structure: communication, daily living, and socialization. The communication scale domain was used as a measure of verbal intelligence.

Although the Leiter-R scale is a test designed for children and adolescents (ages 2–18), it can yield an intelligence quotient (IQ) and a measure of logical ability for all ages. This test provides a non-verbal measure of general intelligence by sampling a wide variety of functions from memory to non-verbal reasoning. A remarkable feature of the Leiter scale is that it can be administered completely without the use of oral language, including instructions, and requires no verbal response from the participant. Because of the exclusion of language, it claims to be more accurate than other tests when testing subjects who cannot or will not provide a verbal response. Leiter contains 20 subtests organized into two domains: visualization and reasoning (VR) and attention and memory (AM). The VR domain is the only domain routinely used at the AGBD Association. Through the different subtests, it is possible to obtain a series of measures connected to intelligence (i.e., reasoning and problem solving).

### Olfactory evaluation

Olfactory function was assessed by means of a standardized test battery, the “Sniffin’ Sticks Extended test” (Burghart Company, Wedel, Germany). One DS subject presented quite severe intellectual disability and reduced speech so that finally, 6 out of 7 DS individuals were able to undergo this assessment.

This validated procedure consists of three subtests, namely, threshold (the concentration at which the odor is reliably detected), discrimination (the subject’s ability to distinguish odors), and identification (the subject has to identify 16 different odors, choosing among different options of answer each time). In order to increase the reliability of the measurements, each subject must give an answer (forced-choice paradigm).

During each subtest, the experimenter removes the pen’s cap and the pen’s tip is held for around 3 s approximately 1 cm under both nostrils. All participants were tested blindfolded by a sleeping mask to prevent visual identification of the odorant-containing pens, for the threshold and discrimination test, as required by the procedure. During the identification assessment for DS people, subjects were asked to choose the answer option that they think to be correct after the odor had been presented, with the possibility to read a paper words list linked to pictures of the four choice options, as previously reported in DS [19].

Scores of the three subtests are presented as a composite “TDI score,” the sum of results obtained for threshold, discrimination, and identification measures. This global score represents a reliable measure to estimate the degree of olfactory function and allows for the detection of normosmia (TDI ≥ 30.3), hyposmia (30.3 > TDI > 16), and functional anosmia (TDI ≤ 16). Kobal et al. introduced the term “functional anosmia” in 2000. This definition means that subjects with a TDI score below 16 are considered completely anosmic or to have some olfactory function left, even if not useful in daily life [32–34]. Indeed, a subject with functional anosmia may still perceive a few odors, be able to discriminate between some of them, or even show olfactory event-related potentials [35]. However, this residual olfactory function does not contribute to the enjoyment of food/drink or to the detection of spoiled food or gas leaks.

### Olfactory swabbing

After olfactory assessment, all participants underwent olfactory swabbing (DS = 7; euploid controls = 10). An expert otolaryngologist explored nasal cavities using a 30° rigid endoscope. Olfactory swabbing sampling was performed using a sterile disposable nasal swab (Copan Flock Technologies, Brescia, Italy) in both nasal cavities. The human olfactory neuroepithelium is located on the cribriform plate, the superior part of the nasal septum, and the superior and middle turbinate [36, 37]. To minimize discomfort in this kind of individuals, the swab was gently rolled on the mucosa surface at the level of the middle turbinate. We previously showed that, from a surface of 2 cm^2^, it is possible to collect ~ 2 × 10^6^ cells for both nostrils, and among these cells, ~ 30% are ONs [20, 22]. This technique is a gentle approach to collect in vivo olfactory epithelial cells and no participant evaluated this procedure as painful. In particular, the most frequent DS individuals’ opinion after the procedure was of “no pain and no discomfort at all.” Swabs were then immersed in RNA stabilization solution for total RNA extraction. Additional swabs were immersed in a fixative solution (Diacyte, Diapath, Italy) for cytological quality control of the samples.

### RNA extraction

Nasal swabs (DS = 7; euploid controls = 10), collected in TRIzol reagent (Invitrogen, Italy), were frozen and stored at − 80 °C. RNA was extracted from each sample using Direct-zol™ RNA Miniprep Plus kit (Zymo Research, CA, USA) following the manufacturer’s instructions. Briefly, the tubes were shaken for 8 s and the swab head removed. Then, each tube was centrifuged at 14,000 × g to remove particular debris and the supernatant was transferred into an RNase-free tube. An equal volume of ethanol (95–100%) was added to each sample and transferred into the columns, and then centrifuged. After two washes, the RNA was eluted. Purification of total RNA was performed using Agencourt RNAClean XP beads (2 × the volume per RNA volume; Beckman Coulter Genomics, Danvers, MA, USA). The concentration and purity of the total RNA samples were measured using the NanoDrop ND-1000 Spectrophotometer (NanoDrop Technologies Inc., Wilmington, DE). RNA integrity was assessed with an Agilent 2100 Bioanalyzer and the RNA 6000 LabChip kit (Agilent Technologies, Palo Alto, CA). Total RNA was then verified on Bioanalyzer 2100 (Agilent Technologies) to assess its quality and integrity, to a final RIN of 6.6 ± 1, and then quantified using Qubit RNA HS assay.

### RNA preparation and sequencing

Samples were further processed with Lexogen RiboCop rRNA depletion kit to remove the ribosomal content and prepared for sequencing using Lexogen SENSE RNA-seq kit following the manufacturer’s protocol (Lexogen). The 17 samples were sequenced by using the Illumina NextSeq 500 applying the 75-single-end chemistry. The data were deposited with links to BioProject accession number PRJNA789170 in the NCBI BioProject and SRA databases.

### RNAseq data analysis

Sequenced reads were trimmed by using cutadapt v1.16 [38] to remove the first 9 nucleotides associated with the library preparation. Trimmed reads were mapped with Salmon v0.9.1 [39] to the Ensembl Homo sapiens GRCh38 cDNA (ftp.ensembl.org/pub/release-92/fasta/homo_sapiens/cdna/Homo_sapiens.GRCh38.cdna.all.fa.gz) using v92 of the Ensembl gene annotation (http://ftp.ensembl.org/pub/release-98/gtf/homo_sapiens/Homo_sapiens.GRCh38.98.gtf.gz). Automatic selection of library type (-l A) and aggregated gene-level abundance estimation (–geneMap) were added to standard salmon parameters. Salmon was executed into a quasi-mapping-based mode (salmon quant), and to improve the read mapping process, whose performance could be reduced by a short read length (~ 66 bp), a k-mers size of 21 was chosen to calculate salmon genome index.

The obtained tables were aggregated into a unique file of raw counts and further normalized to account for sequencing depth between samples, using the procedure implemented in the DESeq2 package [40]. Data analysis, statistical testing, and plotting were performed with Python3 and R, exploiting appropriate libraries and packages.

### Differential expression analysis

Differential expression (DE) analysis was performed with DESeq2 version 1.22.1 [40] with standard parameters. The full DESeq2 pipeline was applied to raw gene counts to characterize DE genes (DEGs). Genes with an adjusted *p*-value lower than 0.1 were considered differentially expressed. No specific filter on the fold change was applied.

### Enrichment analysis

Pathway enrichment analysis was performed on DE genes by using *enrichPathway* function of reactomePA R package [41], exploiting features contained in the Reactome database [42], which includes most of the known biochemical reactions and pathways. *enrichPathway* was applied with default parameters. Differential expressed genes have been used also as input of *enrichGO* function of the Bioconductor package *clusterProfiler* [43]. This function is designed for classifying genes based on GO distribution at a specific level, allowing to select among the three orthogonal ontologies of GO: molecular function (MF), biological process (BP), and cellular component (CC). *enrichGO* was run with default parameters, applying BH *p*-value correction.

#### Correlation analysis

Pearson’s correlation coefficients among the results of the olfactory and cognitive tests and/or the normalized counts of differentially expressed genes were calculated by using rcorr method of the pingouin python package (https://pingouin-stats.org/index.html). Correlations among olfactory scores and normalized gene counts have been calculated considering the two groups together (6 DS and 10 euploid controls) as well as separated by group. Instead, correlation among olfactory scores and cognitive scores was assessed within the DS group. The Benjamini–Hochberg correction [44] for multiple comparisons was used to correct *p*-values and assess the false discovery rate (FDR).

## Results

### Cognitive evaluation

The Vineland II assessment resulted in 6 DS individuals showing severe intellectual disability and 1 DS individual showing moderate intellectual disability. Regarding the Leiter-R assessment, 2 DS individuals showed a moderate, 3 DS individuals a moderate/severe, and 2 DS individuals severe intellectual disability (Table 1).

**Table 1:** Cognitive assessment in DS individuals. Total weighted scores of both Vineland II Communication area and Leiter-R visualization and reasoning domain. *F*, female; *M*: male

DS individuals	Sex	Vineland II Communication	Leiter-R visualization and reasoning
198,365	F	27	10
198,367	F	32	14
198,366	F	34	12
198,364	F	30	9
198,368	M	33	10
232,286	M	37	13
232,331	M	20	7

^*^Total weighted scores

### Olfactory evaluation and olfactory swabbing

In accordance with our previous work [19], all DS individuals (*n* = 6) showed a clear olfactory deficit in all the three assessed domains (Threshold, Discrimination, Identification). In particular, all DS were markedly hyposmic with one case at the limit of functional anosmia (TDI score: 16.5). All euploid controls (*n* = 10) were normosmic (Table 2). Olfactory swabbing was bilaterally performed in all the recruited individuals (7 DS individuals and 10 euploid controls) and all of the obtained samples were of good quality, showing both neuronal and non-neuronal cellular component in both DS and euploid controls at light microscopy check, as previously reported [22].

**Table 2:** Olfactory assessment. TDI (total smell score) and threshold (T), discrimination (D), and identification (I) domain scores in both euploid controls and Down syndrome (DS) individuals (*F*, female; *M*, male). DS subject (code: 232,331) was unable to attend the olfactory evaluation due to quite severe mental retardation and reduced speech but underwent the olfactory swabbing procedure

Code	Sex	TDI score	T	D	I
Euploid controls					
173064	F	34.5	6.5	14	14
173065	M	35.75	8.75	13	14
173066	M	32.75	7.75	10	15
173067	F	41	12	14	15
232280	F	37.25	9.25	15	13
232281	F	31.5	4.5	13	14
232282	M	32.75	7.75	12	13
232283	M	34.5	10.5	12	12
232284	F	33	8	12	13
232285	F	36.5	7.5	13	16
Down syndrome					
198365	F	22.5	11.5	5	6
198367	F	22.5	6.5	8	8
198366	F	21.75	7.75	6	8
198364	F	20.25	1.25	7	12
198368	M	16.5	1.5	5	10
232286	M	22.25	2.25	10	10
232331	M	-	-	-	-

### Sequencing results and DE analysis

To evaluate if significant differences in gene expression among DS individuals and euploid controls were detectable, we sequenced the RNA depleted from rRNA of 17 people (7 DS individuals and 10 euploid controls) by means of Illumina NextSeq 500, after the proper RNA quality check (RIN 6.6 ± 1). For each sample, 66.8 ± 18.3 million reads were produced, with a minimum of 37.9 and a maximum of 91, thus granting a high coverage of sequenced transcripts. Several alignment and feature association pipelines were tested (data not shown), finding the best choice in the transcript-level quantification of salmon, accounting for the percentage of the assigned reads to the features (61.2 ± 5.1).

**Fig. 1:**
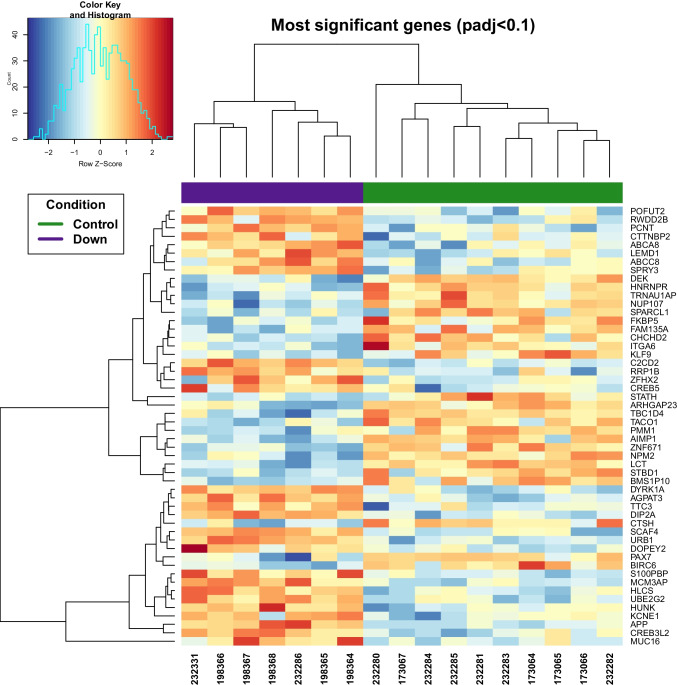
Down syndrome DEG heatmap. Cluster heatmap shows samples in columns and genes in rows. The level of expression is represented by the background color, where blue means low and red means high expression. Experimental conditions are shown in green and purple, illustrating euploid controls and Down syndrome (DS) individuals respectively

Raw count tables were processed using DESeq2 in order to identify genes showing a differential expression (adj *p*-value < 0.1) between controls and DS individuals. A total of 52 differentially expressed genes (DEGs) were detected (Table S1), the majority of which has a |logFC|> 0.5, although no filter on fold change was applied. As expected, several DEGs are located in chromosome 21, and genes such as *APP*, *DYRK1A*, and *DOPEY2* were significantly upregulated in DS individuals (Fig. 1). In addition, we also noticed that the misregulation is spread along the entire genome (Fig. S1): in fact, other interesting DEGs, including *MUC16*, *S100PBP*, *CREB3L2* and *CREB5*, which could play a role in the DS related olfactory peripheral impairment, are located outside of the chromosome 21.

Regarding the downstream steps, no significant result was showed. A possible explanation for the non-significant enrichment analysis results (related to Reactome pathways and GO) could be the relative low number of detected DEGs. Nevertheless, pathway analysis allowed us to better characterize data found in the previous step. At this regard, we noticed modifications of glycosylation processes, mainly O-linked and mucin associated, mediated by *MUC16* and *POFUT2*. In addition, *APP* was found to be involved in different processes so that its upregulation could trigger an increase in inflammation and neuronal dysfunction (Fig. S2; Table S2; Table S3).

### Olfactory test scores and gene correlation analysis

**Fig. 2:**
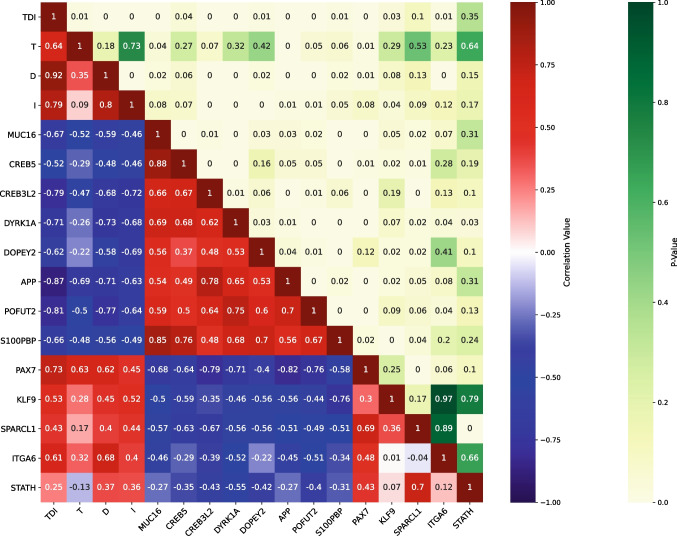
DEG and olfactory scores correlation matrix. Pearson’s coefficient (lower triangle) and *p*-value (upper triangle) between the expression of interesting differentially expressed genes in Down syndrome (DS) individuals (upregulated: *MUC16*, *CREB5*, *CREB3L2*, *DYRK1A*, *DOPEYE2*, *APP*, *POFUT2*, *S100PBP*; downregulated: *PAX7*, *KLF9*, *SPARCL1*, *ITGA6*, *STATH*) and olfactory test scores (TDI, T, D, I) are shown in the matrix. Blue background indicates negative correlation while red indicates positive. *p*-value background ranges from pale yellow (high significance) to green (no significance). T, threshold; D, discrimination; I, identification; TDI, total smell score

Pearson’s analysis showed different significant correlations (Fig. 2). In particular, a subset of interesting genes involved in neuronal function, cellular regeneration, and mucus physiology located in the chromosome 21 and also in chromosomes other than 21 was considered for correlations (i.e., *APP*, *DYRK1A*, *DOPEY2*, *S100PBP*, *CREB3L2*, *CREB5*, *POFUT2*, *MUC16*, *PAX7*, *KLF9*, *SPARCL1*, *ITGA6*, *STATH*). Among the DS upregulated genes (i.e., *APP*, *DYRK1A*, *DOPEY2*, *S100PBP*, *CREB3L2*, *CREB5*, *POFUT2*, *MUC16*), a strong significant negative correlation with the global olfactory TDI score emerged. Thus, when gene expression increases, olfactory performance decreases (*APP ρ* = − 0.87, *p*-value = 0; *MUC16 ρ* = − 0.67, *p*-value = 0; *CREB5 ρ* = − 0.52, *p*-value = 0.04; *CREB3L2 ρ* = − 0.79, *p*-value = 0; *DYRK1A ρ* = − 0.71, *p*-value = 0; *DOPEY2 ρ* = − 0.62, *p*-value = 0.01; *POFUT2 ρ* = − 0.81, *p*-value = 0; *S100PBP ρ* = − 0.66, *p*-value = 0.01). On the other hand, looking at the downregulated genes, a significant positive correlation with the TDI score emerged, namely when gene expression increases, olfactory performance increases. This is particularly clear for the *PAX7* and *ITGA6* genes (PAX7 *ρ* = 0.73, *p*-value = 0; ITGA6 *ρ* = 0.61, *p*-value = 0.01).

Analyzing the single subtest olfactory scores, the correlation between the threshold score and *MUC16* expression (*ρ* = − 0.52, *p-*value = 0.04) is particularly interesting. Regarding discrimination score, a significant correlation is showed both with the *CREB3L2* (*ρ* = − 0.68, *p-*value = 0), *DYRK1A* (*ρ* = − 0.73, *p-*value = 0), *POFUT2* (*ρ* = − 0.77, *p-*value = 0), and *S100PBP* (*ρ* = − 0.56, *p*-value = 0.02) genes. The identification (I) score shows a strong correlation with both the *CREB3L2* (*ρ* = − 0.72, *p-*value = 0), *DYRK1A* (*ρ* = − 0.68, *p-*value = 0), and *DOPEY2* (*ρ* = − 0.62, *p-*value = 0) genes.

Regarding the relationship between downregulated genes and olfactory scores, *PAX7* expression was found to be strongly correlated with both threshold and discrimination (*ρ* = 0.63 and *ρ* = 0.62 respectively, *p*-value = 0.01), while *ITGA6* showed a positive correlation with the discrimination score (*ρ* = 0.68, *p*-value = 0).

In addition, the aforementioned results were also retained by means of the Benjamini/Hochberg FDR method (*p*-value = 0.1) showing a robust significant correlation between olfactory scores and genes’ expression for the global TDI score (*APP ρ* = − 0.87, *p-*value = 0; *MUC16 ρ* = − 0.67, *p-*value = 0.01; *CREB5 ρ* = − 0.52, *p-*value = 0.06; *CREB3L2 ρ* = − 0.79, *p-*value = 0; *DYRK1A ρ* = − 0.71, *p-*value = 0.01; *DOPEY2 ρ* = − 0.62, *p-*value = 0.02; *POFUT2 ρ* = − 0.81, *p-*value = 0; *S100PBP ρ* = − 0.66, *p-*value = 0.02) and for the following single subtests: threshold score and MUC16 expression (*ρ* = − 0.52, *p-*value = 0.06); discrimination and *CREB3L2* (*ρ* = − 0.68, *p-*value = 0.01); discrimination and *DYRK1A* (*ρ* = − 0.73, *p-*value = 0.01); discrimination and *POFUT2* (*ρ* = − 0.77, *p-*value = 0); discrimination and *S100PBP* (*ρ* = − 0.56, *p-*value = 0.05); identification with *CREB3L2* (*ρ* = − 0.72, *p-*value = 0.01), *DYRK1A* (*ρ* = − 0.68, *p-*value = 0.01), and *DOPEY2* (*ρ* = − 0.69, *p-*value = 0.01) genes. The same thing occurs considering the DS downregulated gene *PAX7* and the TDI score (*ρ* = 0.73, *p-*value = 0.01) and *ITGA6* and the TDI score (*ρ* = 0.61, *p-*value = 0.03) as well as for the single subtest scores: both threshold, discrimination with *PAX7* (*ρ* = 0.63 and *ρ* = 0.62 respectively, *p*-value = 0.03), and discrimination with ITGA6 (*ρ* = 0.68, *p-*value = 0.02).

### Olfactory test scores and cognitive evaluation correlation analysis

For correlation analysis, the weighted scores of both cognitive scales were considered (Table 1). For both the Vineland-II Communication scale and the Leiter-R visualization and reasoning domain, non-significant correlation emerged with TDI, T, D, and I scores (Table S4).

## Discussion

To our knowledge, this is the first pilot study that explores gene expression in olfactory neuroepithelium cytological samples of DS individuals compared to euploid controls. Additionally, correlation analysis among olfactory scores and normalized gene counts was calculated as well as among genomic data and cognitive data, the latter only in DS individuals.

It is clear that DS individuals exhibit differential gene expression when compared to euploid controls, even if inter-individual variability is present (Fig. 1). Not all upregulated genes are located in chromosome 21, supporting the previous knowledge that the trisomy 21 *status* induces not only a change in chromosome 21 genes but also whole-genome perturbation, causing a disomic gene misregulation [45–47].

The main findings of the study are as follows: (1) all DS individuals were markedly hyposmic, with one case at the limit of functional anosmia, and a strong correlation emerged between olfactory function and gene expression. In particular, a negative correlation emerged with the DS upregulated genes, namely when gene expression increases, olfactory performance decreases. In addition, a less evident positive correlation emerged with DS downregulated genes, meaning that the expression of such genes is directly related to the olfactory performance. (2) All DS individuals had a moderate to severe cognitive impairment, and through the aforementioned cognitive tests (Vineland-II and Leiter-R), a non-significant correlation emerged between olfactory function and cognition.

The first finding of this study is that olfactory function was severely impaired in DS individuals, for all olfactory domains (i.e., threshold, discrimination, identification), in agreement with our previous results [19]. In addition, this deficit was strongly related to gene expression especially for the upregulated genes (i.e., *APP*, *DYRK1A*, *DOPEY2*, *CREB5*, *CREB3L2*, *MUC16*, *POFUT2*, *S100PBP*). To our knowledge, no study has ever evaluated expression of these genes in the human olfactory neuroepithelium of DS individuals. In particular, some upregulated genes, located in chromosome 21, could be related to neuronal function. At this regard, the well-known *APP* gene (chromosome 21, cytogenetic band 21q 21.3) is relevant to neurite growth, neuronal adhesion, and axonogenesis and the extra copy of *APP* gene could affect the correct olfactory neurons function with synapse signaling disruption and neuroinflammation, as showed in the brain [48]. Indeed, two *post-mortem* morphological studies showed dystrophic neurites and β amyloid deposits in the olfactory mucosa of DS [49, 50] and a preclinical work showed that the expression of a human *APP* mutation in mice impairs connectivity and function of the peripheral olfactory neural circuit, even in the absence of plaques [51].

Another interesting upregulated gene is the *DYRK1A* gene (chromosome 21, cytogenetic band: 21q 22.13) which is located in the DS critical region of chromosome 21 and plays a key role in neurogenesis, outgrowth of axons and dendrites, neuronal trafficking, and aging [52]. Previous preclinical work showed strong expression of *DYRK1A* gene in the olfactory bulb and in the piriform/entorhinal cortex suggesting a possible involvement of *DYRK1A* in the physiology of olfaction [53]. Hence, *DYRK1A* gene upregulation in DS olfactory epithelium could interfere with physiological peripheral olfactory processing, contributing to the olfactory deficit genesis.

*DOPEY2* (chromosome 21, cytogenetic band 21q22.12) is another upregulated gene that could possibly be implicated in correct olfactory neurons function. *DOPEY2* is also located in the DS chromosome 21 critical region and plays a role in the membrane protein trafficking with possible involvement in learning, memory, and intellectual disability pathogenesis [23, 54]. This gene is reported to be upregulated in DS human and trisomic mice tissues [55] with no study involving the olfactory mucosa.

Looking at other upregulated genes located in chromosomes other than 21 and possibly involved in olfactory neuroepithelium physiology, there is the *S100PBP* gene (chromosome 1, cytogenetic band 1p35.1). This gene encodes a protein which is a binding partner of S100 proteins, a large protein family found in a wide range of cells, and involved in the regulation of a number of cellular processes such as cell cycle progression, differentiation, and cellular calcium signaling, the latter playing a meaningful role in olfactory pathway activation [56, 57]. Moreover, within the S100 protein family, it is important to mention that the S100B (encoded by a gene located on the chromosome 21) was shown to be involved in *APP* processing, protein inclusion formation, and tau post-translational modifications in Down syndrome [48]. Therefore, the upregulation of the S100 binding protein gene here observed might interfere with correct *APP* processing, affecting olfaction.

Other extra-21 genes found to be upregulated are the *CREB* genes (*CREB5*, *CREB3L2*) (chromosome 7 cytogenetic band p15.1-p14.3 and q33). These genes encode a well-known transcription factor modulated by cAMP (cyclic AMP-responsive element-binding protein), involved in various pathways [58] and in different cellular processes including neuronal survival and synaptic plasticity [59–62]. Furthermore, the CREB activity was reported to be regulated also by the *DYRK1A* gene [63]. The *CREB* gene was also reported to have a role in the physiological airway mucous cell differentiation [64]. Indeed, olfactory mucosa is covered by a mucus layer involved in multiple protective functions and in the mechanism of odorant detection through different pre-receptor events. Olfactory mucosal enzymes participate in the olfactory signal termination and modulation [65–71]. A recent proteome analysis revealed different proteins in the mucus with a potential involvement in olfaction, correlating with olfactory threshold and identification [72]. Moreover, preclinical studies showed that the *CREB* signaling pathway is necessary for the acquisition of olfactory aversive learning in young rats [73] and that exposure of mice to odorant mixture induced a significant *CREB* signal increase in both olfactory sensory neurons and sustentacular cell nuclei [74].

Other two differently expressed genes are *MUC16* (chromosome 19, cytogenetic band 19p13.2) and *POFUT2* (chromosome 21, cytogenetic band 21q22.3) which upregulation in DS could have implications on the correct mucous cell function and on olfactory cell survival, both meaningful elements for preserving the olfactory function.

On the other hand, among the downregulated genes, interestingly, *ITGA6* (chromosome 2, cytogenetic band 2q31.1) was identified in horizontal basal cells of the olfactory epithelium [75, 76], while the *PAX7* (chromosome 1, cytogenetic band 1p36.13) gene was found in embryonic olfactory precursor cells, contributing to the development of different neuronal and non-neuronal cell lineages [77]. Since olfactory function preservation relies on stem cell activity, thus, the downregulation of these genes in the DS olfactory neuroepithelium might contribute to olfactory deficit genesis.

All DS individuals had cognitive impairment, and through the available Vineland-II Communication and Leiter-R visualization and reasoning domains, non-significant correlation emerged between the cognitive weighted scores and all the olfactory scores. This fact might be related to the small DS sample size and to the olfactory profile revealed, which is very similar in all individuals (i.e., all were markedly hyposmic and one case was at the limit of functional anosmia; see Table 2). In addition, it is important to mention that at the AGBD Association, the Leiter-R attention and memory domains were not used and other more detailed cognitive measurements such as the Wechsler Adult Intelligence Scale-Revised are no longer used because of being time-consuming. Actually, in DS, the memory ability is known to be poor, also with olfactory stimuli, and then, memory function could be meaningful for the required olfactory tasks especially for discrimination and identification tests [19, 78, 79]. Following this line of reasoning, we can assume that the cognitive deficit certainly plays a role in determining the detected olfactory impairment, even if the basic cognitive tests here available failed to uncover the relationship between olfaction and cognition. Hence, to overcome this limit, further studies with a more detailed cognitive measurement are required to compare the DS group with a euploid control group with non-DS individuals but having cognitive disabilities. This would add new information to deepen investigate the possible link between the olfactory deficit and cognition in this syndrome. Nevertheless, based on these data, it might be possible to assume that the known olfactory deficit reported in Down syndrome could be not only attributable to a cortical involvement but also to more peripheral mechanisms.

However, we must consider these results with caution, since very preliminary data on a small sample size. Indeed, this is only a first step investigation, and future studies on a bigger sample size are necessary. It would be also important to assess the protein expression to see if the protein level matches the RNAseq data, better unraveling the link to the emerged olfactory deficit. Moreover, it is important to mention that we analyzed epithelial samples of the olfactory mucosa characterized by a heterogeneous cell population, neuronal and non-neuronal, and a more precise gene expression attribution will be needed in further studies. In addition, in the middle turbinate, olfactory neurons are unevenly distributed, compared to those located in the mucosal surface covering the nasal vault. Moreover, in humans, the olfactory epithelium is not clearly cut from the non-olfactory tissue, in contrast to rodents where there is a marked boundary [80, 81]. Despite the aforementioned limitations, this first exploratory approach gives new insights into the DS olfactory system research, starting from the olfactory neuroepithelium, the first cellular step on the olfactory way. In addition, regarding the sampling technique, olfactory swabbing could represent an innovative gentle technique to study the olfactory epithelium in living DS individuals. Indeed, in our experience, all DS participants referred minimal or no discomfort at all during sampling procedure. This chance might also open to the future research on the peripheral neurodegeneration signs of this genetic syndrome, a peculiar model of early AD-like pathology.

## Supplementary Information

Below is the link to the electronic supplementary material.

Supplementary file1 (PDF 18 KB)

Supplementary file2 (PDF 497 KB)

Supplementary file3 (XLSX 14 KB)

Supplementary file4 (DOCX 36 KB)

Supplementary file5 (XLSX 70 KB)

Supplementary file6 (DOCX 13 KB)

## Data Availability

RNAseq data that support the findings of this study have been deposited in NCBI BioProject and SRA databases (BioProject accession number PRJNA789170).
