# Supplementary figures and images for: RNAseq analysis of olfactory neuroepithelium cytological samples in individuals with Down syndrome compared to euploid controls: a pilot study
Source: Neurol Sci. 2022 Nov 17;44(3):919–30. doi: 10.1007/s10072-022-06500-2 (PMC9925603; doi:10.1007/s10072-022-06500-2)

Number of DE genes

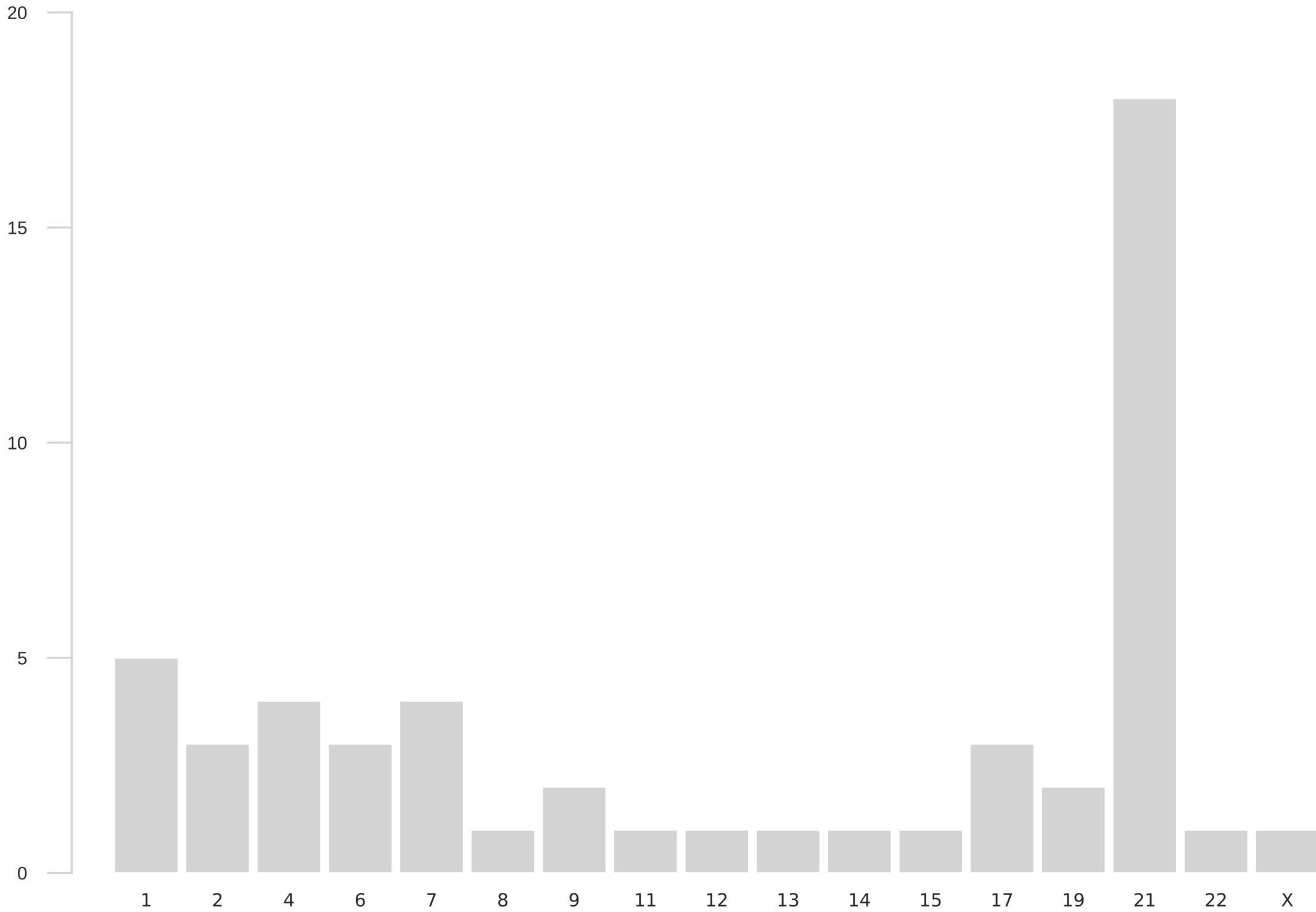

Supplement: Supplementary file 1 — Supplementary file1 (PDF 18 KB) [file 10072_2022_6500_MOESM1_ESM.pdf]

A

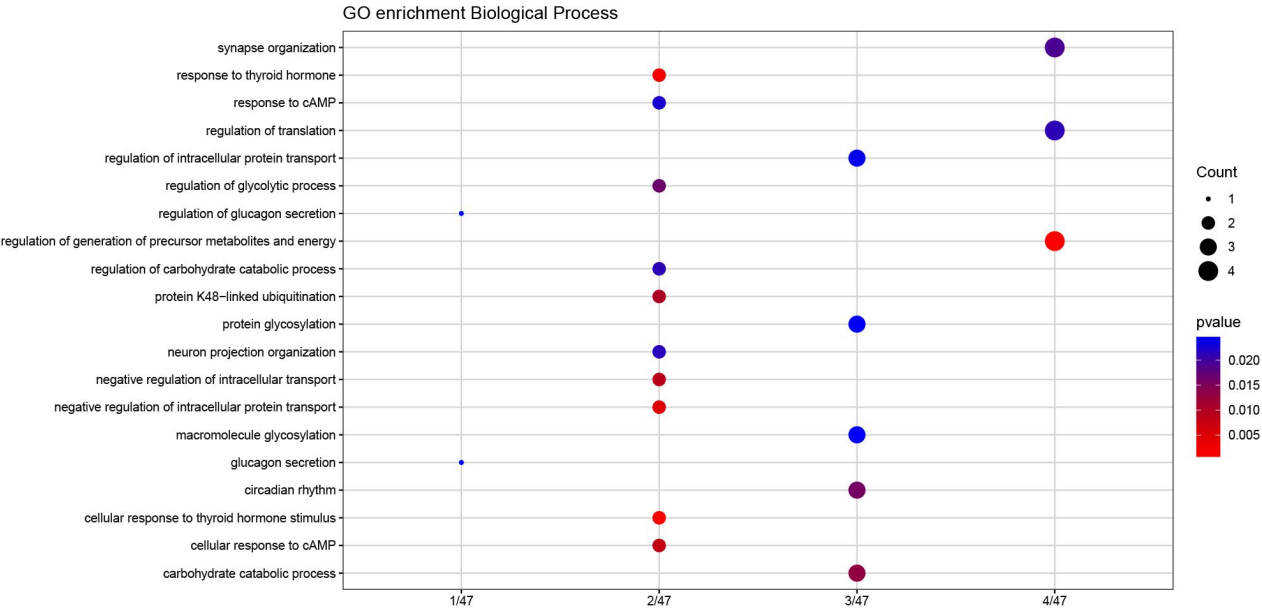

B

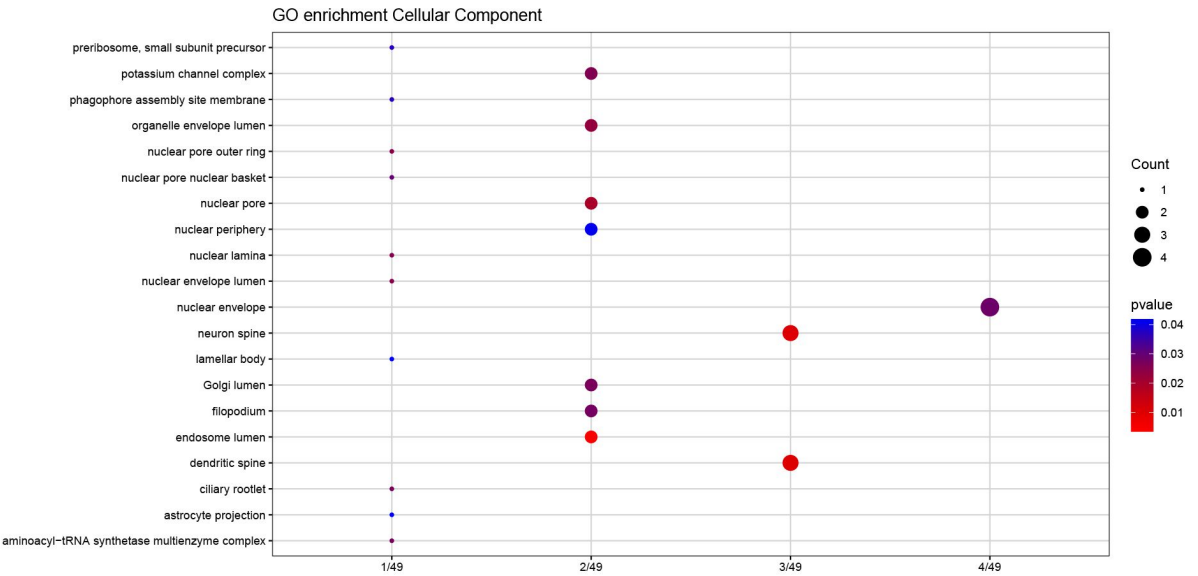

C

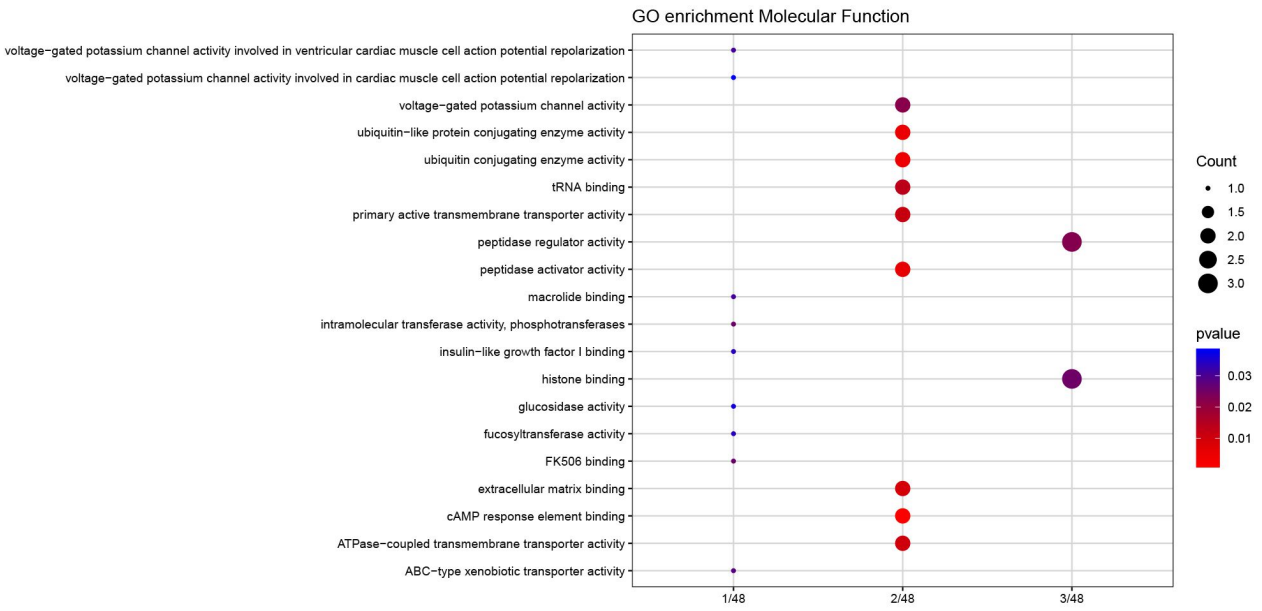

Supplement: Supplementary file 2 — Supplementary file2 (PDF 497 KB) [file 10072_2022_6500_MOESM2_ESM.pdf]
