# Supplementary material for: RNAseq analysis of olfactory neuroepithelium cytological samples in individuals with Down syndrome compared to euploid controls: a pilot study
Source: Neurol Sci. 2022 Nov 17;44(3):919–30. doi: 10.1007/s10072-022-06500-2 (PMC9925603; doi:10.1007/s10072-022-06500-2)
Supplement: Supplementary file 4 — Supplementary file4 (DOCX 36 KB) [file 10072_2022_6500_MOESM4_ESM.docx]

**Table S2**

Pathway enrichment analysis uses a hypergeometric model to assess whether the number of selected genes associated with a reactome pathway is larger than expected. The following table shows the complete list of pathways derived from enrichment analysis results based on DEGs.

ID: pathway ID from Reactome database; Description: full name of the pathway; pvalue: value for statistical significance; p.adjust: adjusted pvalue; geneID, gene name of the enriched genes in the pathway.

| **ID** | **Description** | **pvalue** | **p.adjust** | **geneID** |
| --- | --- | --- | --- | --- |
| **R-HSA-5668914** | Diseases of metabolism | 0.0269 | 0.3644 | HLCS;LCT;MUC16 |
| **R-HSA-189085** | Digestion of dietary carbohydrate | 0.0290 | 0.3644 | LCT |
| **R-HSA-196780** | Biotin transport and metabolism | 0.0290 | 0.3644 | HLCS |
| **R-HSA-446107** | Type I hemidesmosome assembly | 0.0290 | 0.3644 | ITGA6 |
| **R-HSA-8957275** | Post-translational protein phosphorylation | 0.0334 | 0.3644 | APP;SPARCL1 |
| **R-HSA-879415** | Advanced glycosylation endproduct receptor signaling | 0.0342 | 0.3644 | APP |
| **R-HSA-5173105** | O-linked glycosylation | 0.0352 | 0.3644 | MUC16;POFUT2 |
| **R-HSA-5576893** | Phase 2 - plateau phase | 0.0394 | 0.3644 | KCNE1 |
| **R-HSA-844456** | The NLRP3 inflammasome | 0.0419 | 0.3644 | APP |
| **R-HSA-381426** | Regulation of Insulin-like Growth Factor (IGF) transport and uptake by Insulin-like Growth Factor Binding Proteins (IGFBPs) | 0.0436 | 0.3644 | APP;SPARCL1 |
| **R-HSA-5083625** | Defective GALNT3 causes familial hyperphosphatemic tumoral calcinosis (HFTC) | 0.0445 | 0.3644 | MUC16 |
| **R-HSA-5083636** | Defective GALNT12 causes colorectal cancer 1 (CRCS1) | 0.0445 | 0.3644 | MUC16 |
| **R-HSA-9022699** | MECP2 regulates neuronal receptors and channels | 0.0445 | 0.3644 | FKBP5 |
| **R-HSA-5083632** | Defective C1GALT1C1 causes Tn polyagglutination syndrome (TNPS) | 0.0470 | 0.3644 | MUC16 |
| **R-HSA-622312** | Inflammasomes | 0.0547 | 0.3644 | APP |
| **R-HSA-3296482** | Defects in vitamin and cofactor metabolism | 0.0572 | 0.3644 | HLCS |
| **R-HSA-8862803** | Deregulated CDK5 triggers multiple neurodegenerative pathways in Alzheimer's disease models | 0.0572 | 0.3644 | APP |
| **R-HSA-8863678** | Neurodegenerative Diseases | 0.0572 | 0.3644 | APP |
| **R-HSA-8935690** | Digestion | 0.0572 | 0.3644 | LCT |
| **R-HSA-9609523** | Insertion of tail-anchored proteins into the endoplasmic reticulum membrane | 0.0572 | 0.3644 | APP |
| **R-HSA-9613829** | Chaperone Mediated Autophagy | 0.0572 | 0.3644 | PCNT |
| **R-HSA-8980692** | RHOA GTPase cycle | 0.0583 | 0.3644 | STBD1;ARHGAP23 |
| **R-HSA-379716** | Cytosolic tRNA aminoacylation | 0.0622 | 0.3644 | AIMP1 |
| **R-HSA-933542** | TRAF6 mediated NF-kB activation | 0.0622 | 0.3644 | APP |
| **R-HSA-977068** | Termination of O-glycan biosynthesis | 0.0622 | 0.3644 | MUC16 |
| **R-HSA-210991** | Basigin interactions | 0.0648 | 0.3644 | ITGA6 |
| **R-HSA-9660826** | Purinergic signaling in leishmaniosis infection | 0.0673 | 0.3644 | APP |
| **R-HSA-9664424** | Cell recruitment (pro-inflammatory response) | 0.0673 | 0.3644 | APP |
| **R-HSA-1538133** | G0 and Early G1 | 0.0698 | 0.3644 | DYRK1A |
| **R-HSA-3000170** | Syndecan interactions | 0.0698 | 0.3644 | ITGA6 |
| **R-HSA-5621480** | Dectin-2 family | 0.0698 | 0.3644 | MUC16 |
| **R-HSA-8963743** | Digestion and absorption | 0.0698 | 0.3644 | LCT |
| **R-HSA-9615933** | Postmitotic nuclear pore complex (NPC) reformation | 0.0698 | 0.3644 | NUP107 |
| **R-HSA-9609507** | Protein localization | 0.0699 | 0.3644 | APP;CHCHD2 |
| **R-HSA-5683826** | Surfactant metabolism | 0.0747 | 0.3644 | CTSH |
| **R-HSA-3000157** | Laminin interactions | 0.0772 | 0.3644 | ITGA6 |
| **R-HSA-8866652** | Synthesis of active ubiquitin: roles of E1 and E2 enzymes | 0.0772 | 0.3644 | UBE2G2 |
| **R-HSA-5619115** | Disorders of transmembrane transporters | 0.0790 | 0.3644 | NUP107;ABCC8 |
| **R-HSA-168271** | Transport of Ribonucleoproteins into the Host Nucleus | 0.0797 | 0.3644 | NUP107 |
| **R-HSA-168333** | NEP/NS2 Interacts with the Cellular Export Machinery | 0.0797 | 0.3644 | NUP107 |
| **R-HSA-170822** | Regulation of Glucokinase by Glucokinase Regulatory Protein | 0.0797 | 0.3644 | NUP107 |
| **R-HSA-5619107** | Defective TPR may confer susceptibility towards thyroid papillary carcinoma (TPC) | 0.0797 | 0.3644 | NUP107 |
| **R-HSA-168274** | Export of Viral Ribonucleoproteins from Nucleus | 0.0846 | 0.3644 | NUP107 |
| **R-HSA-180746** | Nuclear import of Rev protein | 0.0846 | 0.3644 | NUP107 |
| **R-HSA-180910** | Vpr-mediated nuclear import of PICs | 0.0846 | 0.3644 | NUP107 |
| **R-HSA-445989** | TAK1 activates NFkB by phosphorylation and activation of IKKs complex | 0.0846 | 0.3644 | APP |
| **R-HSA-159227** | Transport of the SLBP independent Mature mRNA | 0.0871 | 0.3644 | NUP107 |
| **R-HSA-165054** | Rev-mediated nuclear export of HIV RNA | 0.0871 | 0.3644 | NUP107 |
| **R-HSA-4085377** | SUMOylation of SUMOylation proteins | 0.0871 | 0.3644 | NUP107 |
| **R-HSA-5663084** | Diseases of carbohydrate metabolism | 0.0871 | 0.3644 | LCT |
| **R-HSA-1296065** | Inwardly rectifying K+ channels | 0.0895 | 0.3644 | ABCC8 |
| **R-HSA-159230** | Transport of the SLBP Dependant Mature mRNA | 0.0895 | 0.3644 | NUP107 |
| **R-HSA-3301854** | Nuclear Pore Complex (NPC) Disassembly | 0.0895 | 0.3644 | NUP107 |
| **R-HSA-432720** | Lysosome Vesicle Biogenesis | 0.0895 | 0.3644 | APP |
| **R-HSA-9615710** | Late endosomal microautophagy | 0.0895 | 0.3644 | PCNT |
| **R-HSA-176033** | Interactions of Vpr with host cellular proteins | 0.0919 | 0.3644 | NUP107 |
| **R-HSA-177243** | Interactions of Rev with host cellular proteins | 0.0919 | 0.3644 | NUP107 |
| **R-HSA-3232142** | SUMOylation of ubiquitinylation proteins | 0.0968 | 0.3644 | NUP107 |
| **R-HSA-5173214** | O-glycosylation of TSR domain-containing proteins | 0.0968 | 0.3644 | POFUT2 |
| **R-HSA-8864260** | Transcriptional regulation by the AP-2 (TFAP2) family of transcription factors | 0.0968 | 0.3644 | DEK |
| **R-HSA-1483166** | Synthesis of PA | 0.1016 | 0.3644 | AGPAT3 |
| **R-HSA-168276** | NS1 Mediated Effects on Host Pathways | 0.1016 | 0.3644 | NUP107 |
| **R-HSA-68877** | Mitotic Prometaphase | 0.1025 | 0.3644 | NUP107;PCNT |
| **R-HSA-159231** | Transport of Mature mRNA Derived from an Intronless Transcript | 0.1041 | 0.3644 | NUP107 |
| **R-HSA-159234** | Transport of Mature mRNAs Derived from Intronless Transcripts | 0.1065 | 0.3644 | NUP107 |
| **R-HSA-379724** | tRNA Aminoacylation | 0.1065 | 0.3644 | AIMP1 |
| **R-HSA-9696273** | RND1 GTPase cycle | 0.1065 | 0.3644 | FAM135A |
| **R-HSA-168325** | Viral Messenger RNA Synthesis | 0.1089 | 0.3644 | NUP107 |
| **R-HSA-9646399** | Aggrephagy | 0.1089 | 0.3644 | PCNT |
| **R-HSA-9012999** | RHO GTPase cycle | 0.1130 | 0.3697 | STBD1;FAM135A;ARHGAP23 |
| **R-HSA-4615885** | SUMOylation of DNA replication proteins | 0.1136 | 0.3697 | NUP107 |
| **R-HSA-4570464** | SUMOylation of RNA binding proteins | 0.1184 | 0.3781 | NUP107 |
| **R-HSA-6811436** | COPI-independent Golgi-to-ER retrograde traffic | 0.1278 | 0.3781 | AGPAT3 |
| **R-HSA-2980766** | Nuclear Envelope Breakdown | 0.1301 | 0.3781 | NUP107 |
| **R-HSA-9013405** | RHOD GTPase cycle | 0.1301 | 0.3781 | STBD1 |
| **R-HSA-191859** | snRNP Assembly | 0.1325 | 0.3781 | NUP107 |
| **R-HSA-194441** | Metabolism of non-coding RNA | 0.1325 | 0.3781 | NUP107 |
| **R-HSA-3371497** | HSP90 chaperone cycle for steroid hormone receptors (SHR) | 0.1371 | 0.3781 | FKBP5 |
| **R-HSA-72203** | Processing of Capped Intron-Containing Pre-mRNA | 0.1377 | 0.3781 | NUP107;HNRNPR |
| **R-HSA-168643** | Nucleotide-binding domain. leucine rich repeat containing receptor (NLR) signaling pathways | 0.1418 | 0.3781 | APP |
| **R-HSA-6784531** | tRNA processing in the nucleus | 0.1418 | 0.3781 | NUP107 |
| **R-HSA-3000171** | Non-integrin membrane-ECM interactions | 0.1464 | 0.3781 | ITGA6 |
| **R-HSA-2022090** | Assembly of collagen fibrils and other multimeric structures | 0.1509 | 0.3781 | ITGA6 |
| **R-HSA-8986944** | Transcriptional Regulation by MECP2 | 0.1532 | 0.3781 | FKBP5 |
| **R-HSA-446219** | Synthesis of substrates in N-glycan biosythesis | 0.1555 | 0.3781 | PMM1 |
| **R-HSA-913709** | O-linked glycosylation of mucins | 0.1555 | 0.3781 | MUC16 |
| **R-HSA-1268020** | Mitochondrial protein import | 0.1578 | 0.3781 | CHCHD2 |
| **R-HSA-3906995** | Diseases associated with O-glycosylation of proteins | 0.1668 | 0.3781 | MUC16 |
| **R-HSA-3371453** | Regulation of HSF1-mediated heat shock response | 0.1690 | 0.3781 | NUP107 |
| **R-HSA-380259** | Loss of Nlp from mitotic centrosomes | 0.1713 | 0.3781 | PCNT |
| **R-HSA-380284** | Loss of proteins required for interphase microtubule organization from the centrosome | 0.1713 | 0.3781 | PCNT |
| **R-HSA-4551638** | SUMOylation of chromatin organization proteins | 0.1735 | 0.3781 | NUP107 |
| **R-HSA-70171** | Glycolysis | 0.1735 | 0.3781 | NUP107 |
| **R-HSA-1169408** | ISG15 antiviral mechanism | 0.1757 | 0.3781 | NUP107 |
| **R-HSA-1445148** | Translocation of SLC2A4 (GLUT4) to the plasma membrane | 0.1757 | 0.3781 | TBC1D4 |
| **R-HSA-199992** | trans-Golgi Network Vesicle Budding | 0.1757 | 0.3781 | APP |
| **R-HSA-8854518** | AURKA Activation by TPX2 | 0.1779 | 0.3781 | PCNT |
| **R-HSA-9013408** | RHOG GTPase cycle | 0.1801 | 0.3781 | STBD1 |
| **R-HSA-159236** | Transport of Mature mRNA derived from an Intron-Containing Transcript | 0.1823 | 0.3781 | NUP107 |
| **R-HSA-2995410** | Nuclear Envelope (NE) Reassembly | 0.1823 | 0.3781 | NUP107 |
| **R-HSA-3000178** | ECM proteoglycans | 0.1845 | 0.3781 | APP |
| **R-HSA-3108214** | SUMOylation of DNA damage response and repair proteins | 0.1867 | 0.3781 | NUP107 |
| **R-HSA-5619084** | ABC transporter disorders | 0.1867 | 0.3781 | ABCC8 |
| **R-HSA-422356** | Regulation of insulin secretion | 0.1889 | 0.3781 | ABCC8 |
| **R-HSA-446193** | Biosynthesis of the N-glycan precursor (dolichol lipid-linked oligosaccharide. LLO) and transfer to a nascent protein | 0.1889 | 0.3781 | PMM1 |
| **R-HSA-8852135** | Protein ubiquitination | 0.1911 | 0.3781 | UBE2G2 |
| **R-HSA-1474244** | Extracellular matrix organization | 0.1915 | 0.3781 | APP;ITGA6 |
| **R-HSA-1169410** | Antiviral mechanism by IFN-stimulated genes | 0.1933 | 0.3781 | NUP107 |
| **R-HSA-168928** | DDX58/IFIH1-mediated induction of interferon-alpha/beta | 0.1954 | 0.3781 | APP |
| **R-HSA-9663891** | Selective autophagy | 0.1954 | 0.3781 | PCNT |
| **R-HSA-380270** | Recruitment of mitotic centrosome proteins and complexes | 0.1976 | 0.3781 | PCNT |
| **R-HSA-380287** | Centrosome maturation | 0.1976 | 0.3781 | PCNT |
| **R-HSA-72202** | Transport of Mature Transcript to Cytoplasm | 0.2019 | 0.3781 | NUP107 |
| **R-HSA-168142** | Toll Like Receptor 10 (TLR10) Cascade | 0.2041 | 0.3781 | APP |
| **R-HSA-168176** | Toll Like Receptor 5 (TLR5) Cascade | 0.2041 | 0.3781 | APP |
| **R-HSA-216083** | Integrin cell surface interactions | 0.2041 | 0.3781 | ITGA6 |
| **R-HSA-975871** | MyD88 cascade initiated on plasma membrane | 0.2041 | 0.3781 | APP |
| **R-HSA-5628897** | TP53 Regulates Metabolic Genes | 0.2084 | 0.3781 | TACO1 |
| **R-HSA-2565942** | Regulation of PLK1 Activity at G2/M Transition | 0.2105 | 0.3781 | PCNT |
| **R-HSA-9013404** | RAC2 GTPase cycle | 0.2105 | 0.3781 | STBD1 |
| **R-HSA-3371556** | Cellular response to heat stress | 0.2126 | 0.3781 | NUP107 |
| **R-HSA-1474290** | Collagen formation | 0.2147 | 0.3781 | ITGA6 |
| **R-HSA-9616222** | Transcriptional regulation of granulopoiesis | 0.2147 | 0.3781 | DEK |
| **R-HSA-446728** | Cell junction organization | 0.2169 | 0.3781 | ITGA6 |
| **R-HSA-5250924** | B-WICH complex positively regulates rRNA expression | 0.2169 | 0.3781 | DEK |
| **R-HSA-70326** | Glucose metabolism | 0.2169 | 0.3781 | NUP107 |
| **R-HSA-975138** | TRAF6 mediated induction of NFkB and MAP kinases upon TLR7/8 or 9 activation | 0.2169 | 0.3781 | APP |
| **R-HSA-975155** | MyD88 dependent cascade initiated on endosome | 0.2190 | 0.3781 | APP |
| **R-HSA-168181** | Toll Like Receptor 7/8 (TLR7/8) Cascade | 0.2211 | 0.3781 | APP |
| **R-HSA-168164** | Toll Like Receptor 3 (TLR3) Cascade | 0.2232 | 0.3781 | APP |
| **R-HSA-380320** | Recruitment of NuMA to mitotic centrosomes | 0.2232 | 0.3781 | PCNT |
| **R-HSA-381119** | Unfolded Protein Response (UPR) | 0.2232 | 0.3781 | CREB3L2 |
| **R-HSA-9013423** | RAC3 GTPase cycle | 0.2232 | 0.3781 | STBD1 |
| **R-HSA-141424** | Amplification of signal from the kinetochores | 0.2273 | 0.3781 | NUP107 |
| **R-HSA-141444** | Amplification of signal from unattached kinetochores via a MAD2 inhibitory signal | 0.2273 | 0.3781 | NUP107 |
| **R-HSA-168138** | Toll Like Receptor 9 (TLR9) Cascade | 0.2273 | 0.3781 | APP |
| **R-HSA-5619102** | SLC transporter disorders | 0.2315 | 0.3781 | NUP107 |
| **R-HSA-5620912** | Anchoring of the basal body to the plasma membrane | 0.2315 | 0.3781 | PCNT |
| **R-HSA-166166** | MyD88-independent TLR4 cascade | 0.2336 | 0.3781 | APP |
| **R-HSA-937061** | TRIF(TICAM1)-mediated TLR4 signaling | 0.2336 | 0.3781 | APP |
| **R-HSA-166058** | MyD88:MAL(TIRAP) cascade initiated on plasma membrane | 0.2377 | 0.3781 | APP |
| **R-HSA-168188** | Toll Like Receptor TLR6:TLR2 Cascade | 0.2377 | 0.3781 | APP |
| **R-HSA-9645723** | Diseases of programmed cell death | 0.2398 | 0.3781 | APP |
| **R-HSA-1296071** | Potassium Channels | 0.2418 | 0.3781 | ABCC8 |
| **R-HSA-382556** | ABC-family proteins mediated transport | 0.2418 | 0.3781 | ABCA8 |
| **R-HSA-611105** | Respiratory electron transport | 0.2418 | 0.3781 | TACO1 |
| **R-HSA-9020702** | Interleukin-1 signaling | 0.2418 | 0.3781 | APP |
| **R-HSA-168179** | Toll Like Receptor TLR1:TLR2 Cascade | 0.2439 | 0.3781 | APP |
| **R-HSA-181438** | Toll Like Receptor 2 (TLR2) Cascade | 0.2439 | 0.3781 | APP |
| **R-HSA-5250913** | Positive epigenetic regulation of rRNA expression | 0.2479 | 0.3793 | DEK |
| **R-HSA-5578749** | Transcriptional regulation by small RNAs | 0.2479 | 0.3793 | NUP107 |
| **R-HSA-72306** | tRNA processing | 0.2500 | 0.3799 | NUP107 |
| **R-HSA-163685** | Integration of energy metabolism | 0.2520 | 0.3804 | ABCC8 |
| **R-HSA-977225** | Amyloid fiber formation | 0.2580 | 0.3870 | APP |
| **R-HSA-69618** | Mitotic Spindle Checkpoint | 0.2620 | 0.3905 | NUP107 |
| **R-HSA-9610379** | HCMV Late Events | 0.2680 | 0.3968 | NUP107 |
| **R-HSA-9648025** | EML4 and NUDC in mitotic spindle formation | 0.2700 | 0.3972 | NUP107 |
| **R-HSA-2408522** | Selenoamino acid metabolism | 0.2719 | 0.3976 | AIMP1 |
| **R-HSA-196849** | Metabolism of water-soluble vitamins and cofactors | 0.2817 | 0.4033 | HLCS |
| **R-HSA-2132295** | MHC class II antigen presentation | 0.2817 | 0.4033 | CTSH |
| **R-HSA-9707564** | Cytoprotection by HMOX1 | 0.2836 | 0.4033 | TACO1 |
| **R-HSA-2500257** | Resolution of Sister Chromatid Cohesion | 0.2875 | 0.4033 | NUP107 |
| **R-HSA-163200** | Respiratory electron transport. ATP synthesis by chemiosmotic coupling. and heat production by uncoupling proteins. | 0.2894 | 0.4033 | TACO1 |
| **R-HSA-114608** | Platelet degranulation | 0.2933 | 0.4033 | APP |
| **R-HSA-1483206** | Glycerophospholipid biosynthesis | 0.2933 | 0.4033 | AGPAT3 |
| **R-HSA-1500931** | Cell-Cell communication | 0.2933 | 0.4033 | ITGA6 |
| **R-HSA-5576891** | Cardiac conduction | 0.2933 | 0.4033 | KCNE1 |
| **R-HSA-162909** | Host Interactions of HIV factors | 0.2952 | 0.4033 | NUP107 |
| **R-HSA-8856688** | Golgi-to-ER retrograde transport | 0.3009 | 0.4033 | AGPAT3 |
| **R-HSA-76005** | Response to elevated platelet cytosolic Ca2+ | 0.3028 | 0.4033 | APP |
| **R-HSA-166016** | Toll Like Receptor 4 (TLR4) Cascade | 0.3047 | 0.4033 | APP |
| **R-HSA-168273** | Influenza Viral RNA Transcription and Replication | 0.3047 | 0.4033 | NUP107 |
| **R-HSA-9609690** | HCMV Early Events | 0.3047 | 0.4033 | NUP107 |
| **R-HSA-1632852** | Macroautophagy | 0.3065 | 0.4033 | PCNT |
| **R-HSA-68886** | M Phase | 0.3079 | 0.4033 | NUP107;PCNT |
| **R-HSA-202733** | Cell surface interactions at the vascular wall | 0.3084 | 0.4033 | ITGA6 |
| **R-HSA-211000** | Gene Silencing by RNA | 0.3103 | 0.4033 | NUP107 |
| **R-HSA-162599** | Late Phase of HIV Life Cycle | 0.3122 | 0.4033 | NUP107 |
| **R-HSA-5663220** | RHO GTPases Activate Formins | 0.3140 | 0.4033 | NUP107 |
| **R-HSA-446652** | Interleukin-1 family signaling | 0.3159 | 0.4033 | APP |
| **R-HSA-5621481** | C-type lectin receptors (CLRs) | 0.3177 | 0.4033 | MUC16 |
| **R-HSA-68875** | Mitotic Prophase | 0.3177 | 0.4033 | NUP107 |
| **R-HSA-3781865** | Diseases of glycosylation | 0.3196 | 0.4034 | MUC16 |
| **R-HSA-212165** | Epigenetic regulation of gene expression | 0.3306 | 0.4127 | DEK |
| **R-HSA-453279** | Mitotic G1 phase and G1/S transition | 0.3306 | 0.4127 | DYRK1A |
| **R-HSA-9612973** | Autophagy | 0.3342 | 0.4150 | PCNT |
| **R-HSA-162587** | HIV Life Cycle | 0.3360 | 0.4150 | NUP107 |
| **R-HSA-168255** | Influenza Infection | 0.3431 | 0.4216 | NUP107 |
| **R-HSA-168898** | Toll-like Receptor Cascades | 0.3449 | 0.4216 | APP |
| **R-HSA-9609646** | HCMV Infection | 0.3502 | 0.4236 | NUP107 |
| **R-HSA-9711123** | Cellular response to chemical stress | 0.3502 | 0.4236 | TACO1 |
| **R-HSA-6798695** | Neutrophil degranulation | 0.3692 | 0.4442 | STBD1;CTSH |
| **R-HSA-1428517** | The citric acid (TCA) cycle and respiratory electron transport | 0.3813 | 0.4563 | TACO1 |
| **R-HSA-3108232** | SUMO E3 ligases SUMOylate target proteins | 0.3880 | 0.4615 | NUP107 |
| **R-HSA-72163** | mRNA Splicing - Major Pathway | 0.3896 | 0.4615 | HNRNPR |
| **R-HSA-9013149** | RAC1 GTPase cycle | 0.3929 | 0.4631 | ARHGAP23 |
| **R-HSA-2990846** | SUMOylation | 0.3979 | 0.4652 | NUP107 |
| **R-HSA-196854** | Metabolism of vitamins and cofactors | 0.4011 | 0.4652 | HLCS |
| **R-HSA-2467813** | Separation of Sister Chromatids | 0.4028 | 0.4652 | NUP107 |
| **R-HSA-72172** | mRNA Splicing | 0.4028 | 0.4652 | HNRNPR |
| **R-HSA-69275** | G2/M Transition | 0.4108 | 0.4691 | PCNT |
| **R-HSA-397014** | Muscle contraction | 0.4124 | 0.4691 | KCNE1 |
| **R-HSA-453274** | Mitotic G2-G2/M phases | 0.4140 | 0.4691 | PCNT |
| **R-HSA-913531** | Interferon Signaling | 0.4156 | 0.4691 | NUP107 |
| **R-HSA-375276** | Peptide ligand-binding receptors | 0.4188 | 0.4691 | APP |
| **R-HSA-5617833** | Cilium Assembly | 0.4188 | 0.4691 | PCNT |
| **R-HSA-6811442** | Intra-Golgi and retrograde Golgi-to-ER traffic | 0.4204 | 0.4691 | AGPAT3 |
| **R-HSA-1483257** | Phospholipid metabolism | 0.4360 | 0.4842 | AGPAT3 |
| **R-HSA-416476** | G alpha (q) signalling events | 0.4421 | 0.4886 | APP |
| **R-HSA-8939211** | ESR-mediated signaling | 0.4527 | 0.4979 | FKBP5 |
| **R-HSA-162906** | HIV Infection | 0.4689 | 0.5132 | NUP107 |
| **R-HSA-68882** | Mitotic Anaphase | 0.4718 | 0.5132 | NUP107 |
| **R-HSA-2555396** | Mitotic Metaphase and Anaphase | 0.4732 | 0.5132 | NUP107 |
| **R-HSA-9658195** | Leishmania infection | 0.4972 | 0.5367 | APP |
| **R-HSA-76002** | Platelet activation. signaling and aggregation | 0.5094 | 0.5473 | APP |
| **R-HSA-72766** | Translation | 0.5457 | 0.5821 | AIMP1 |
| **R-HSA-69620** | Cell Cycle Checkpoints | 0.5494 | 0.5821 | NUP107 |
| **R-HSA-71387** | Metabolism of carbohydrates | 0.5507 | 0.5821 | NUP107 |
| **R-HSA-1852241** | Organelle biogenesis and maintenance | 0.5519 | 0.5821 | PCNT |
| **R-HSA-9006931** | Signaling by Nuclear Receptors | 0.5556 | 0.5834 | FKBP5 |
| **R-HSA-446203** | Asparagine N-linked glycosylation | 0.5617 | 0.5871 | PMM1 |
| **R-HSA-983168** | Antigen processing: Ubiquitination & Proteasome degradation | 0.5676 | 0.5907 | UBE2G2 |
| **R-HSA-418594** | G alpha (i) signalling events | 0.5782 | 0.5990 | APP |
| **R-HSA-195258** | RHO GTPase Effectors | 0.5886 | 0.6070 | NUP107 |
| **R-HSA-373076** | Class A/1 (Rhodopsin-like receptors) | 0.5976 | 0.6135 | APP |
| **R-HSA-3700989** | Transcriptional Regulation by TP53 | 0.6296 | 0.6435 | TACO1 |
| **R-HSA-71291** | Metabolism of amino acids and derivatives | 0.6387 | 0.6499 | AIMP1 |
| **R-HSA-983169** | Class I MHC mediated antigen processing & presentation | 0.6417 | 0.6501 | UBE2G2 |
| **R-HSA-112316** | Neuronal System | 0.6730 | 0.6789 | ABCC8 |
| **R-HSA-449147** | Signaling by Interleukins | 0.7172 | 0.7203 | APP |
| **R-HSA-500792** | GPCR ligand binding | 0.7211 | 0.7211 | APP |
