# Supplementary material for: RNAseq analysis of olfactory neuroepithelium cytological samples in individuals with Down syndrome compared to euploid controls: a pilot study
Source: Neurol Sci. 2022 Nov 17;44(3):919–30. doi: 10.1007/s10072-022-06500-2 (PMC9925603; doi:10.1007/s10072-022-06500-2)
Supplement: Supplementary file 6 — Supplementary file6 (DOCX 13 KB) [file 10072_2022_6500_MOESM6_ESM.docx]

| **Smell tests** | **Vineland II**  **Communication** | **Leiter-R**  **Visualization and reasoning** |
| --- | --- | --- |
| Threshold (T) | r=-052 | r=0.15 |
| Discrimination (D) | r=0.64 | r=0.65 |
| Identification (I) | r=0.36 | r=-0.29 |
| TDI score | r=-0.07 | r=0.54 |

**Table S4.** Pearson’s correlation (coefficient r) between olfactory test scores and Vineland II Communication v-scale scores (total weighted scores) as well as between the olfactory test scores and Leiter-R total weighted scores in DS individuals. All p values were non-significant.
